# Asymptomatic *Plasmodium falciparum* infection is associated with anaemia in pregnancy and can be more cost-effectively detected by rapid diagnostic test than by microscopy in Kinshasa, Democratic Republic of the Congo

**DOI:** 10.1186/1475-2875-13-132

**Published:** 2014-04-02

**Authors:** Junior R Matangila, Jean Lufuluabo, Axel L Ibalanky, Raquel A Inocêncio da Luz, Pascal Lutumba, Jean-Pierre Van Geertruyden

**Affiliations:** 1 Département de Médecine Tropicale, Université de Kinshasa, B.P. 747, Kinshasa, XI, République Démocratique du Congo; 2 Institut Superieur de Techniques Médicales, Kinshasa RDC, Kinshasa, République Démocratique du Congo; 3 International Health Unit, Department of Epidemiology, University of Antwerp, Campus DrieEiken, Universiteitsplein 1, Wilrijk 2610, Belgium

**Keywords:** Asymptomatic *P. falciparum* infection, Anaemia, Pregnancy, Cost-effectiveness, Democratic Republic of the Congo

## Abstract

**Background:**

In areas of high malaria transmission, *Plasmodium falciparum* infection during pregnancy is characterized by malaria-related anaemia, placental malaria and does not always result in clinical symptoms. This situation is associated with poor pregnancy outcomes. The aim of this study was to determine the extent of asymptomatic *P. falciparum* infection, its relation with anaemia as well as the most cost-effective technique for its diagnosis in healthy pregnant women living in Kinshasa, Democratic Republic of the Congo.

**Methods:**

In a cross-sectional study design, information on socio-demographic characteristics and cost data were collected in healthy pregnant women attending antenatal care consultations. *Plasmodium falciparum* infection was diagnosed using rapid diagnostic test (RDT), microscopy and polymerase chain reaction (PCR). Haemoglobin concentration was also determined.

**Results:**

In total, 332 pregnant women were enrolled. RDT and microscopy data were available for all the blood samples and 166 samples were analysed by PCR. The prevalence of asymptomatic *P. falciparum* infection using microscopy, RDTs and PCR, were respectively 21.6%, 27.4% and 29.5%. Taking PCR as a reference, RDTs had a sensitivity of 81.6% and a specificity of 94.9% to diagnose asymptomatic *P. falciparum* infection. The corresponding values for microscopy were 67.3% and 97.4%. The prevalence of anaemia was 61.1% and asymptomatic malaria increased five times the odds (p < 0.001) of having anaemia. RDTs were more cost-effective compared to microscopy. Incremental cost-effectiveness ratio was US$ 63.47 per microscopy adequately diagnosed case.

**Conclusion:**

These alarming results emphasize the need to actively diagnose and treat asymptomatic malaria infection during all antenatal care visits. Moreover, in DRC, malaria and anaemia control efforts should be strengthened by promoting the use of insecticide-treated nets, intermittent preventive treatment with sulphadoxine-pyrimethamine and iron and folic acid supplements.

## Background

Malaria infection in pregnant women is a major problem in sub-Saharan Africa (SSA) with significant risks for the mother and their offspring. Malaria in pregnancy is characterized by a secondary anaemia and the presence of parasites in the placenta known as placental malaria, leading to low birth weight, source of perinatal morbidity and mortality. In areas of high *P. falciparum* transmission, where a high proportion of the population is semi-immune, most pregnant women with malaria are asymptomatic [1-4].

The sequestration of infected erythrocytes in the intervillous spaces induces a local inflammation and a massive infiltration of immune cells (macrophages, monocytes and lymphocytes) and is often observed and described as ‘inflammatory placental malaria’ [2,5]. This condition disturbs exchanges between the mother and the foetus and leads to poor pregnancy outcomes, such as abortion, stillbirths, low birth weight and infant mortality [6]. Annually, up to 200,000 infant deaths are attributed to malaria during pregnancy [7,8]. Moreover, untreated asymptomatic malaria evolves in a chronic infection characterized by marked dyserythropoietic changes in the red cell precursors and increased erythrophagocytosis. It has been suggested that these changes may be mediated, at least in part, by high levels of tumour necrosis factor (TNF) [9-11]. Maternal anaemia consequently increases the incidence of maternal death during pregnancy or post-partum, and is associated with increased foetal and infant mortality, prematurity and low birth weight across many populations [12]. In malaria-endemic areas, it is estimated that 25% of pregnant women are infected with malaria parasites, with the greatest risk of infection and morbidity in primiparous adolescents, and those co-infected with HIV [13].

Diagnosis of asymptomatic *P. falciparum* infection during pregnancy is, therefore, essential to prevent poor pregnancy outcomes, but presents a great challenge. The absence of clinical signs decreases the chances of a clinical diagnosis and a management guided by a decisional algorithm. Moreover, microscopic diagnosis is often challenged by fluctuating and very low parasite densities, due to placental parasite sequestration, the most common form of asymptomatic malaria during pregnancy [14]. Previous studies showed that RDTs detecting *P. falciparum* histidine-rich protein 2 (HRP2) are more sensitive than microscopy and appear to be reliable predictors of adverse outcomes of malaria in pregnancy [15]. However, more recent studies have brought conflicting results [16]. While RDTs have been extensively evaluated in malaria among the non-pregnant population, there are few published data on the performance and cost-effectiveness of RDT for diagnosing asymptomatic *P. falciparum* infection during pregnancy in malaria-endemic areas.

In the Democratic Republic of the Congo (DRC), previous studies have evaluated the prevalence of malaria and placental infection [17], the practice of intermittent preventive treatment (IPTp) [18] and the use of insecticide-treated net (ITN) [19] in pregnant women. However, there is very little data on asymptomatic *P. falciparum* infection and its contribution to anaemia among pregnant women. The latest studies were conducted in 1988 and 2003 and reported a prevalence of malaria infection among women attending antenatal clinics (ANCs) to be respectively 22% and 49% [20,21]. These prevalence estimates in endemic settings were done by microscopy and may have been different if other methods, such as RDT (Rapid Diagnostic Test) and PCR (Polymerase Chain Reaction), had been used [22].

Therefore, this study determined through microscopy, RDTs and PCR the prevalence of asymptomatic *P. falciparum* infection during pregnancy and its relation to anaemia. In addition, their cost and cost-effectiveness to diagnose asymptomatic *P. falciparum* infection during pregnancy, in semi-rural setting in Kinshasa, were assessed.

## Methods

This study was conducted at the Centre Hospitalier de Kingasani II (CHK), commonly known as Maternité des Soeurs. With twenty to thirty deliveries daily, it is the most frequented Centre in Kinshasa and countrywide. Located in the heart of the semi-rural areas of the south-eastern suburbs of Kinshasa, the maternity provides invaluable health care at an accessible rate.

In a cross-sectional survey, the prevalence of asymptomatic *P. falciparum* infection was determined in apparently healthy pregnant women, going to the CHK for their first antenatal care (ANC) visit. Data and blood samples were collected prior to any routine administration of intermittent preventive treatment (IPT). Participants were recruited from July 27^th^, 2012 to August 27^th^, 2012. Women not providing written informed consent or presenting fever, muscle aches or other symptoms suggestive for malaria were excluded. Women who received anti-malarial treatment within the past two weeks were also excluded from the study. A structured questionnaire was used to obtain information on age, parity, and gestational age, level of education, previous or current use of anti-malarial drugs and the use of bed nets.

### Blood sample collection

Blood samples were collected from a finger prick for laboratory analysis which included: thick blood smears for microscopy, RDT performance, determination of haemoglobin concentration and molecular analysis. For molecular analysis, blood was collected on a filter paper (Whatmann 3 MM), dried thoroughly, put in individual zip lock plastic bags containing desiccant and stored at room temperature (< 25°C) until completion of the study and then transported to the Tropical Diseases Research Centre (TDRC) in Zambia for molecular analyses.

### Laboratory analysis

Microscopy, RDTs and PCR were used to detect *P. falciparum* infection. Giemsa-stained thick blood smears (TBS) were used for microscopy. Blood slides were examined using light microscopy at 1,000 × magnification. Hundred microscopic fields were examined in the thick smear before concluding that a blood slide was negative. All slides were read twice by experienced microscopists. If the discrepancy was greater than 15%, a third reader was used to confirm diagnosis. The parasite density per microlitre of blood was computed using the following formula: (Number of trophozoites × 6,000)/Number of leucocytes. Besides microscopy, rapid diagnostic tests (RDTs) were also performed. In this study the SD Bioline Malaria Ag Pf® detecting HRP2 was used.

### Plasmodium species-specific diagnostic PCR assay

As microscopy in pregnancy has a lower sensitivity due to placental sequestering of parasites and RDTs have internal validity problems, species specific PCR was conducted on all microscopy negative samples but RDT positive samples. In addition, 166 randomly chosen samples were analysed using nested Plasmodium species diagnostic PCR assay. Molecular analysis was performed at TDRC Ndola, Zambia.

The blood spot samples of 5 mm diameter size were soaked in 0.5% saponin in phosphate-buffered saline (PBS), incubated for 10 minutes at room temperature in a 1.5-ml tube, and centrifuged at 14000 rpm. The supernatant was discarded and blood spots were washed in 1 ml of PBS. One hundred Fifty microlitres of a 2% Chelex-100 resin work solution (Bio-Rad Richmond, CA) and 50 μl of water (pH 9.5) were added to the sample in a1.5-ml tube and incubated at 100°C for 10 minutes. After centrifugation at 10,000 g for 1 min, the supernatant was collected and stored at -20°C, prior to the PCR assay.

### Polymerase chain reaction investigation

Nested PCR assay was performed as a two-step procedure. Firstly, amplification of Plasmodium genus specific fragment was carried out as follow: PCR mixture containing buffer, dNTPs, MgCl2, primers, Taq polymerases and sterile water. All PCR reactions were carried out in a total volume of 20 μl. One μl of the purified template DNA was used for the first reaction, in which the fragment spanned by rPLU5 (5' CCTGTT GTTGCCTTAAACTTC 3') and rPLU6 (5' TTAAAATTGTT GCAGTTAAAACG 3') was amplified. Secondly, a 1 μl aliquot from the product of the first PCR reaction was subsequently used as a template for *Plasmodium falciparum*-specific fragment amplification using FAL1 (5' TTAAACTGGTTTGGGAAAACCAAATATATT3') and FAL2 (5’ ACACAATGAACTCAA TCATGACTACCCGTC 3') specific primers [23]. Positive and negative controls were always included in the assays. A negative control without DNA template and a positive control with appropriate template (3D7) were always included.

PCR products were detected by running 20 μl of DNA product on a 3% agarose gel, small fragments (Eurogentec®), which was subsequently stained with a 0.5 μg/ml ethidium bromide solution and visualized under ultraviolet transillumination. The specific size of the PCR product (second amplification) was 205 bp for *P. falciparum.*

### Haemoglobin concentration

Determining the concentration of haemoglobin was performed with a portable HemoControl® device. Anaemia was defined by a haemoglobin concentration <11 g/dL. Anaemia was classified as severe anaemia Hb <7 g/dl, moderate anaemia Hb: 7–9.9 g/dl and mild anaemia Hb: 10-10.9 g/dl. [24].

### Statistical analysis

The population of pregnant women in the health zone of Kingasani was estimated at 7,187 [25]. With an expected malaria prevalence of 30%, a desired accuracy set at 5% and a confidence interval of 95%, the minimum sample size was calculated at 309 pregnant women.

Data was entered and stored in Epi info™7. Descriptive statistics were employed for the analysis of socio-demographic data. Frequencies were used to assess the prevalence of asymptomatic malaria and anaemia in pregnant women. The χ2 test was used to investigate associations between categorical variables. Odds ratios (ORs), 95% confidence intervals (CIs) were calculated and p < 0.05 values were considered to be statistically significant. Multivariate logistic regression models were constructed to identify factors associated with asymptomatic malaria or anaemia during pregnancy. Based on a priori knowledge and a P value less than 0.05 considered as significant, the following variables: Age, sex, parity, marital status, ownership of bed net, presence of lattice on windows, malaria infection and geophagia were included in the model. Backward regression technique was used to construct the model. Statistical analyses were performed using SPSS statistical program, version 17 (SPSS, Chicago, IL, USA).

The sensitivity, specificity, positive predictive value (PPV) and negative predictive value (NPV) of microscopy and RDT (SD Bioline malaria Ag Pf®) were determined with PCR as gold standard, for the 166 samples (50%) of which PCR results were available.

### Collection of data to estimate the cost of *P. falciparum* infection diagnosis

Financial data were obtained by conducting interviews with Hospital managers and laboratory staff in Kingasani health Zone. Costs were collected in Congolese Francs (CDF) and converted to US dollars (exchange rate US$1 = CDF 920, July 2012). The cost for one thick blood smear includes: a glass slide, cotton, lancet, Giemsa and other stains, immersion oil, alcohol and gloves. The personnel cost for the service provided corresponded to the time spent from drawing blood samples, preparing the thick and thin smears, staining, reading and reporting the results. At CHK II as in all public maternities in Kinshasa, RDT kits are donated and the personnel service is free of charge. In the present study, for RDT diagnostic strategy, the effective contact time with those seeking care, which accounted for the personnel cost, and the cost of RDT kit were the main input parameters to figure out the amount of money a pregnant women would pay. The effective contact time was comprised of: drawing blood samples from patients, applying samples onto the test, test reading and reporting of results. The time was recorded on a “time sheet” by laboratory personnel for every RDT and microscopy service provided. Laboratory staff work eight hours from Monday to Friday and 5 hours on Saturday, thus 68 hours (4,080 minutes) weekly. Assuming a month of four weeks; laboratory staff work 272 hours (16,320 minutes) monthly. Staff salary data were provided by the staff manager.

### Ethical considerations

This study was approved by the Ethical Committee board of the University of Kinshasa. Test results of the thick blood smear were revealed to all participants three days after sample collection. All women were given mebendazole, iron and folic acid on a routine basis and were treated with SP regardless of the laboratory test results.

## Results

### Socio-demographic characteristics of the pregnant women

In total, 332 pregnant women from 18 to 39 years old agreed to participate in the study. The median age was 27 years (interquartile range (IQR) = 22-33), and 26% were primigravidae, the median gravidity was 3 (IQR = 1-5). Eighty one percent of the subjects were married and 66.2% had not completed secondary school. Thirty-seven percent (37%) of the pregnant women had started their antenatal care in the third trimester. The mean gestational age in primigravidae at the time of the first prenatal visit was 21.5 weeks (SD ± 4.2) *vs.* 23.6 weeks (SD ± 5.8) in multigravida (p < 0.01) (Table 1).

**Table 1:** **Predictors for asymptomatic *P. falciparum *** **infection and anaemia in asymptomatic pregnant women in semi-rural suburbs of Kinshasa, 2012**

			**Asymptomatic *P. falciparum***					**Anaemia**				
***Socio-demographic characteristic***		**N**	**Positive TBS**	**OR**	**P value**	**AOR 95% CI**	**P value**	**Anaemia**	**OR**	**P value**	**AOR**	**P value**
Age	<20	47(14.2)	24(51.1)	5.2	<0.001	3.2 (1.2-8.2)	0.02*	34(72.3)	1.8	0.09	0.9 (0.3-2.4)	0.8
	20-49	285(85.8)	48(16.9)	1				169(59.3)	1			
Gravidity	Primigravidae	86(25.9)	27(31.4)	2.0	0.01	0.8 (0.3-1.7)	0.5	59(68.6)	1.5	0.1	1.5 (0.8-2.9)	0.2
	Multigravidae	246(74.1)	45(18.3)	1				144(58.5)	1			
Gestational age	1^st^ et 2^ème^ trimester	209(63.0)	42(20.1)	0.8	0.4	0.7 (0.4-1.3)	0.3	134(61.8)	1.1	0.7	1.1 (0.6-1.8)	0.7
	3^rd^ trimester	123(37.0)	30(24.4)	1				69(60.0)	1			
Marital Status°	Married	259(80.9)	41(15.8)	0.22	<0.001	0.3 (0.2-0.8)	0.01*	153(59.1)	0.7	0.2	1.3 (0.6-2.9)	0.6
	Single	61(19.1)	28(45.9)	1				41(67.2)	1			
Ownership and use of bed net	Yes and used	117(35.2)	13(11.1)	0.4	0.002	0.4 (0.2-0.7)	0.005*	72(61.5)	1			
	Yes not used	26(7.8)	10(38.5)	1.8	0.2	1.9 (0.8-4.9)	0.2	17(65.4)	1	0.8	0.9 (0.3-2.4)	0.8
	No	189(56.9)	49(25.9)	1				114(60.3)	1.2	0.7	0.7 (0.5-1.2)	0.2
Presence of lattice on windows	Yes	55(16.6)	13(23.6)	1.1	0.7	1.1 (0.5-2.5)	0.7	36(65.5)	1.2	0.5	1.6 (0.5-4.4)	0.4
	No	277(83.4)	59(21.3)	1				164(60.3)	1			
Malaria	Yes	72(21.7 )	NA					60 (83.3)	4.1	<0.001*	5 (2.3-10.1)	<0.001*
	No	260(78.3 )	NA					143(55.0)	1			
Geophagia	Yes	180 (54.2)	NA					103(57.2)	0.7	0.1	0.7 (0.5-1.2)	0.2
	No	152 (45.8)	NA					100(65.8)	1			

NA: Not applicable *Significant at p < 0.05, OR- odds ratio, AOR- adjusted odds ratio, C.I- confidence interval. °N = 320.

### Prevalence of asymptomatic *P. falciparum* infection in pregnant women at the first prenatal care (PNC) visit

RDT and microscopy data were available for 332 blood samples and of these, 166 samples were analysed by PCR. The prevalence of asymptomatic *P. falciparum* infection using microscopy, RDTs and PCR, were respectively 21.6% (95% CI:17.4-26.6%), 27.4% (95% CI:22.5-32.6%) and 29.5% (95% CI:22.7-37.1%). *Plasmodium malariae* was diagnosed in five women by microscopy, but was not confirmed by PCR. The median parasite density was 126/μl (IQR:105-162). In bivariate analyses, age, gravidity, marital status and the use of bed nets demonstrated a significant association with asymptomatic malaria. The regression model shows that being younger than 20 years old was an independent risk factor associated with asymptomatic *P. falciparum* infection (AOR 3.2;95% CI:1.2-8.2, p = 0.02), while being married (AOR 0.3;95% CI:0.2-0.8, p = 0.01) and spending the night under a bed net (AOR 0.4; 95% CI:0.2-0.7, p < 0.01) was associated with low prevalence of asymptomatic *P. falciparum* infection (Table 1).

### Prevalence of anaemia

The mean haemoglobin concentration was 10.5 ± 1.4 g/dL and the prevalence of anaemia was 61.1% (95% CI:55.7-66.4). Of these anaemic pregnant women, 70 (21.1%), 132 (39.7%), 1 (0.3%), had respectively, mild, moderate and severe anaemia. In multivariate analyses, asymptomatic malaria increased five times the likelihood of having anaemia (AOR 5 95% CI:2.3-10.1; p < 0.001) (Tables 1 and 2).

**Table 2:** Prevalence of anaemia in pregnant women in semi-rural suburbs of Kinshasa, 2012 (N = 332)

			**Percentage**	**Median (Parasite density)**
Haemoglobin concentration	Severe anaemia <7 g/dl	1	0.3%	128 (IQR: 128-128)
	Moderate anaemia 7–9.9 g/dl	135	40.7%	100 (IQR: 78-132)
	Slight anaemia 10-10.9 g/dl	67	20.2%	127 (IQR: 103-159)
	Non-anaemic ≥ 11 g/dl	129	38.9%	120 (IQR: 83-135)

### Possession and use of insecticide-treated nets

Of all participants, 43.1% (95% CI:37.7-48.6%) possessed a bed net. Of these, 69.8% stated that their nets were treated with insecticide and 81.8% reported having slept under the bed net the night before the interview (Table 1).

### PCR confirmation of the RDT positive but microscopy negative cases

**Figure 1:**
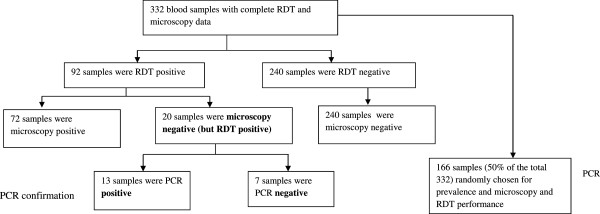
Flow diagram for malaria diagnosis and subsequent PCR analysis in semi-rural suburbs of Kinshasa, 2012.

Out of 332 samples, 92 were RDT positive and 72 were microscopy positive. All 72 microscopy positive samples were confirmed by RDT. Analysis of the 20 samples that were microscopy negative but RDT positive revealed that 65% (13/20) were positive by PCR (95% CI: 40.8- 84.6) (Figure 1).

### Validity of microscopy and RDT using species specific PCR as golden standard

PCR was performed on 166 random chosen samples. Using PCR as the gold standard, microscopy and RDTs had a sensitivity of respectively 67.3% (95% CI:52.5-80.1) and 81.6% (95% CI:68.0-91.2) to diagnose asymptomatic *P. falciparum* infection. Specificity was 97.4% (95% CI:92.7-99.5) and 94.9% (95% CI:89.2-98.1) for microscopy and RDTs, respectively. RDTs demonstrated a positive predictive value (PPV) of 87.0% (95% CI:73.7-95.1%) and a negative predictive value (NPV) of 92.5% (95% CI:86.2-96.5%). The corresponding values for microscopy were 91.7% (95% CI:77.5-98.2%) and 87.7% (95% CI:80.8-92.8%). Microscopy combined to RDT demonstrated a sensitivity of 81.6% (95% CI:68.0-91.2), a specificity of 94.9% (95% CI:89.2-98.1), a PPV of 87.0% (95% CI:73.7-95.1%) and NPV) of 92.5% (95% CI:86.2-96.5%). The differences were not significant for any comparison (Table 3).

**Table 3:** Comparison of RDT and microscopy performance using PCR as golden standard in semi-rural suburbs of Kinshasa, 2012 (N = 166)

		**PCR results**			**Performances**			
		**Positive**	**Negative**	**Total**	**Sensitivity**	**Specificity**	**PPV**	**NPV**
Microscopy	+	33	3	36	67.3% (52.5-80.1)	97.4% (92.7-99.5)	91.7% (77.5-98.2)	87.7% (80.8-92.8)
	-	16	114	130				
RDT	+	40	6	46	81.6% (68.0-91.2)	94.9% (89.2-98.1)	87.0% (73.7-95.1)	92.5% (86.2-96.5)
	-	9	111	120				
Microscopy and RDT	+	40	6	46	81.6% (68.0-91.2)	94.9% (89.2-98.1)	87.0% (73.7-95.1)	92.5% (86.2-96.5)
	-	9	111	120				

### Cost-effectiveness of RDT and microscopy

On the basis of the 166 samples for which PCR results were available, malaria RDT was the most effective with the number of cases correctly diagnosed being 40 (90.9%) compared to 33(88.6%) for microscopy (Table 4). The corresponding proportion of patients correctly diagnosed was 90.9% and 88.6%, respectively. Routine use of RDT would result into an additional 2.3% of patients correctly diagnosed in comparison to microscopy. Thus, incremental cost-effectiveness ratio was US$ 63.47 per adequately diagnosed case for microscopy when compared to RDT strategy (Table 5).

**Table 4:** Comparison of effectiveness of the two diagnostic strategies (N = 166)

**Tests results**	**Diagnostic strategy**	
	**RDT**	**Microscopy**
True positive	40	33
False positive	6	3
True negative	111	114
False negative	9	16
Total sample analysed	166	166
Number correctly diagnosed	151	147
Proportion correctly diagnosed	90.9	88.6

**Table 5:** Results of the cost-effectiveness analysis of the microscopy compared to RDT , Kingasani health zone, Kinshasa, 2013

**Diagnostic strategy**	**Cost (US$)**	**Additional cost (US$)**	**Effectiveness**	**Additional effectiveness**	**ICER (US$)**
Microscopy	2.62	1.46	0.886		63.47
RDT	1.16		0.909	0.023	

ICER: Incremental Cost-effectiveness Ratio.

## Discussion

In the most frequented maternity in Kinshasa, located in the heart of the semi-rural areas Kinshasa, the prevalence of asymptomatic malaria at the first antenatal visit was 27% and 21%, using respectively RDT and microscopy. PCR showed that almost one third and one fifth were missed by microscopy and RDT respectively. Microscopy results (21%) were similar to the 22% value reported in 1988 [20].

These values are paradoxically lower than the 42% found in Lubumbashi, an area of low malaria transmission [21]. Asymptomatic infection is a characteristic in areas of high malaria transmission; therefore, one would expect a higher prevalence in Kinshasa. It can be argued that the prevalence of asymptomatic *P. falciparum* infection may have been somewhat lower, because the study was carried out during the dry season, period subjected to a mild/low malaria transmission. Therefore, another survey during the rainy season may be informative.

Parasite density was also found to be very low in all infected pregnant women. *P. falciparum*-infected but asymptomatic individuals tend to have low and submicroscopic parasite densities [26,27]. Moreover, during pregnancy placental sequestration of parasitized red blood cells may lead to the absence or low grade of peripheral parasitaemia [28].

Other studies also found a protective effect of bed nets against malaria in pregnancy [29]. Other studies have shown that younger women may be more susceptible than older women to malaria because they are still in the process of acquiring natural immunity to pregnancy related malaria [28]. Single women were more likely to have asymptomatic *P. falciparum* infection compared to married women. In addition, owning a mosquito net was associated with being married, as previously reported by Pettifor *et al.* in 2008 [19]. This indicates that marriage or living with a partner, may offer women an ideal setting to promote maternal health. Gravidity did not show any association with asymptomatic *P. falciparum* infection. This indicates that malaria endemicity may be lower than expected impairing the development of immunity against pregnancy-related malaria during successive pregnancies. Similar observations were made in other low malaria-endemic areas [30-33], stressing the need for further studies to assess the endemicity in the DRC. An individual predictor impairing acquisition of immunity is HIV infection [34]. It is, however, very unlikely that this would have affected the present results, as HIV prevalence in ANC in Kinshasa is estimated at 3.9% [35].

Pregnant women were coming for their first antenatal visit at the time of enrolment, thus prior to any administration of IPTp with SP. However *P. falciparum* infection in early pregnancy has been associated with adverse outcomes such as low birth weight LBW [33]. The use of bed nets would, therefore, be a suitable option to protect pregnant women during this period. Almost half of the surveyed women 43% declared to own a bed net. These results are higher than the 26%, 33% and 7% previously reported by UNDP in 2009 [35], Pettifor *et al.* in 2008 [19] and the Programme National de Lutte contre le Paludisme (PNLP) in 2007 [36]. However, only 35% of all pregnant women slept under a bed net, which may suggest a slight awareness of malaria during pregnancy in this specific group [19].

High prevalence of anaemia was observed and strongly correlated with asymptomatic *P. falciparum* infection. This prevalence is similar to those reported in Kisangani in 2000 (65%) and Lubumbashi (in average 65%) in 2003 [37,21]. However, Kinshasa, Kisangani and Lubumbashi belong to three different epidemiological malaria transmission patterns. These findings and the fact that a high proportion of non-malaria infected women were anaemic suggest the presence of other risk factors for anaemia in pregnant women, although the contribution of malaria seems to be significant. The aim of the study was not to assess the risk factors for anaemia in pregnant women; a dedicated study would be more appropriate to disclose this useful information. There was no significant difference in the density of parasitaemia in those with mild, moderate and severe anaemia (Table 2). A similar observation was also reported from Nigeria in 2009 [38]. A limitation of this study was that HIV infection, which is also known to cause anaemia [39], was not documented. Considering HIV when constructing the regression model could have provided more accurate information on the contribution of asymptomatic malaria.

In the present study, no significant difference was found between microscopy and RDT using PCR as the gold standard. This observation was also made elsewhere [16,40]. Combination results of RDTs and microscopy did not improve the sensitivity, specificity, PPV as well as NPV to diagnose asymptomatic *P. falciparum* infection. In addition, all the RDT positive but microscopy negative samples were analysed by PCR and it showed that 65% were in fact submicroscopic infections. The PCR negative samples could be either explained by the persistence of HRP-2 circulation in the blood more than two weeks even after successful clearance of infected erythrocytes in the bloodstream or by the sequestration of the parasites in the placenta while HRP-2 circulate [41]. PCR showed that almost one third and one fifth were missed by microscopy and RDT respectively. These finding highlight the necessity of strengthening IPTp in pregnancy.

This study demonstrated that RDT technique was more cost-effective than microscopy. This finding is supported by others studies carried out in other settings [42,43]. In malaria microscopy, the major cost input was the personnel salary and laboratory equipment. The major parameter that determined the cost of RDT-based strategy was cost of the test. The study did not consider the capital costs of setting up the infrastructure for microscopy diagnosis, which is known to be costly, as well as personnel training from which data were not available (Table 6). If considered, microscopy costs would be higher. Studies have shown that preventive efforts (IPT with SP and insecticide-treated bed net) still leave a large proportion of women with peripheral parasitaemia which is associated with anaemia and impaired delivery outcomes [44]. This highlights the necessity of assessing other strategies such as prompt screening and treatment of women when they present to antenatal care. Therefore, RDT use may be the best available diagnostic alternative for asymptomatic malaria screening in remote or semi-rural endemic areas where infrastructure for microscopy diagnosis is not in place, or where high accuracy of microscopy cannot be assured like in Kinshasa [45].

**Table 6:** **Cost components and unit costs considered for monthly asymptomatic *P*. *falciparum *** **diagnosis, at CHK II, in Kingasani Health Zone, Kinshasa in 2013**

**Items**	**Unit cost (US$)**	**Microscopy strategy (US$) (n = 332)**	**RDT strategy (US$) (n = 332)**
**Exams and supplies**			
Thick blood smear^a^	0.72	239.04	-
SD Bioline Malaria Antigen Pf® - one test	0.79	-	262.28
**Salary**			
Laboratory technician salary	304.34 (monthly)	588.18	123.82
**Equipment**			
Microscope- one unit – value	520.82 ( annual)	43.4 (monthly)	-
Training*			
Microscopy - one annual course	-	-	-
RDT-one annual course	-	-	-
Total		870.62	386.1

^a^Corresponds to the individual cost of an examination, which includes a glass slide, Giemsa and other stains (all components of stains), oil immersion, lancet, cotton, alcohol and gloves.

*Training costs were not available.

## Conclusion

This study reports a high prevalence of anaemia among pregnant women in the health zone of Kingasani, Kinshasa and anaemia is strongly correlated to asymptomatic *P. falciparum* infection. These results are quite alarming and emphasize the need to actively diagnose and treat asymptomatic malaria infection using appropriate techniques during ANC visits in areas of high malaria transmission, regardless of IPTp. On the other hand, though the use of ITNs in DRC is slightly increasing, the promotion of ITN use should be further encouraged. The present study suggests that RDTs may replace blood smears screening of asymptomatic *P. falciparum* infection in pregnancy in some malaria-endemic settings by virtue of their ease-to-use, ability to detect sub-microscopic infection and cost-effectiveness compared with microscopy.

## Abbreviations

AOR: Adjusted Odds-ratio; CI: Confidence Interval; DRC: Democratic Republic of the Congo; IQR: Inter Quartile Range; OR: Odds-ratio; PCR: Polymerase Chain Reaction; PNLP: Programme National de Lutte contre le Paludisme; PRDT: Rapid Diagnostic Test; SSA: Sub-Saharan Africa; TBS: Thick Blood Smear.

## Competing interests

The authors declare that they have no competing interest.

## Author’s contributions

JMR participated in the conception and design of the study protocol, conducted the study, draft the manuscript and participated in molecular analysis. AI and L participated in the design of the study protocol and conducted the study. RIL, PL, JPV reviewed the manuscript and provided critical inputs. All authors read and approved the final manuscript.
